# Activation of mesolimbic reward system via laterodorsal tegmental nucleus and hypothalamus in exercise-induced hypoalgesia

**DOI:** 10.1038/s41598-018-29915-4

**Published:** 2018-08-01

**Authors:** Katsuya Kami, Fumihiro Tajima, Emiko Senba

**Affiliations:** 1 Department of Rehabilitation Medicine, Wakayama Medical University, 811-1 Kimiidera, Wakayama City, Wakayama 641-8509 Japan; 2 Department of Physical Therapy, Osaka Yukioka College of Health Science, 1-1-41 Sojiji, Ibaraki City, Osaka 567-0801 Japan

## Abstract

Ventral tegmental area (VTA) dopamine (DA) neurons are the primary source of dopamine in target structures that constitute the mesolimbic reward system. Previous studies demonstrated that voluntary wheel running (VWR) by neuropathic pain (NPP) model mice produces exercise-induced hypoalgesia (EIH), and that activation of mesolimbic reward system may lead to EIH. However, the neuronal mechanism by which the mesolimbic reward system is activated by VWR is unknown. Here, we found that VWR produces EIH effects and reverses the marked reduction in activated lateral VTA (lVTA)-DA neurons induced by NPP. The proportions of activated laterodorsal tegmental nucleus (LDT)-cholinergic and lateral hypothalamus-orexin neurons were significantly enhanced by VWR. Retrograde tracing and dual immunostaining revealed that VWR activates lVTA-projecting LDT-cholinergic/non-cholinergic and lateral hypothalamic area (LHA)-orexin/non-orexin neurons. Therefore, EIH effects may be produced, at least in part, by activation of the mesolimbic reward system via activation of LDT and LHA neurons.

## Introduction

Physical inactivity or a sedentary lifestyle is known as a risk factor for many diseases, including cardiovascular disease, diabetes, cancer, depression, dementia and chronic pain, whereas physical exercise, such as running, swimming and cycling, is approved as an effective non-pharmacological intervention to improve pain^1,2^. Many studies have demonstrated using neuropathic pain (NPP) and inflammatory pain (IFP) model animals that physical exercise significantly improves pain-related behaviors such as mechanical allodynia and heat hyperalgesia (exercise-induced hypoalgesia: EIH)^3^. Furthermore, multiple events, including marked alterations in cytokines, neurotrophins, neurotransmitters, and endogenous opioids in injured peripheral nerves, dorsal root ganglia, spinal dorsal horns and the brainstem following physical exercise have been proposed as potential mechanisms of EIH effects^3^. However, few studies have focused on the central nervous system to elucidate the mechanisms underlying EIH effects even though chronic pain patients exhibit severe dysfunction in multiple brain regions related to perception, emotion, cognition and behavior^4^.

Ventral tegmental area (VTA) dopamine (DA) neurons are the primary source of dopamine in target structures, such as the medial prefrontal cortex (mPFC) and nucleus accumbens (NAc), which constitute the mesolimbic reward system. The VTA-DA neurons play a central role in motivation and reward processing, which are regulated through inputs from multiple brain regions, including the lateral habenular nucleus (LHb), lateral hypothalamus (LH) and laterodorsal tegmental nucleus (LDT)^5^. A recent study found that the excitability of VTA-DA neurons projecting to the NAc markedly decreases in NPP model mice, whereas optogenetic stimulation of VTA-DA neurons produces analgesic effects^6^. The levels of DA, kappa-opioid receptor, dopamine receptor-D1A and -D2 in the NAc were reduced in inflammatory and NPP model animals^7,8^. Furthermore, human imaging studies indicate that chronic pain leads to a significant impairment of mesolimbic dopamine activity, which may underlie a key system mediating depression with chronic pain^9–13^. Thus, the mesolimbic reward system is a neuronal pathway that can control chronic pain, and activation of the VTA-DA neurons in particular may be effective in improving and preventing chronic pain.

Herrera *et al*.^14^ demonstrated that voluntary wheel running (VWR) and forced treadmill running enhanced activation of both DA neurons in the VTA and FosB expression in the NAc. Specific activation of the VTA-DA neurons and neural pathway from the VTA to NAc markedly increased the amount of VWR^15^. Greenwoods *et al*.^16^ reported that VWR in rats enhances the mRNA level of tyrosine hydroxylase (TH), a rate-limiting enzyme for DA, and increases expression of FosB in the NAc. In addition, it is well-known that rats and mice preferentially select wheel running^14,17^. These findings suggest that similar with feeding and drug abuse, physical exercise is a rewarding behavior for rodents.

A recent study demonstrated that specific inhibition of VTA-DA neurons projecting to the NAc attenuates EIH effects in NPP model mice that ran on a treadmill^18^. In addition, based on our preliminary findings, the VWR improves pain behaviors and increases both TH production and the number of phosphorylated cAMP response element binding protein (pCREB)-expressing VTA-DA neurons in NPP model mice^19^. These results indicate that activation of the mesolimbic reward system following physical exercise may be involved in EIH effects. However, neuronal mechanisms by which physical exercise activates VTA-DA neurons are unknown. VTA-DA neurons were reported to receive excitatory inputs from cholinergic and glutamatergic neurons in the LDT and from orexin neurons in the lateral hypothalamic area (LHA)^5,20^. Therefore, we hypothesized that the LDT and/or LHA neurons activated by physical exercise may play a role in activating VTA-DA neurons, which may consequently produce EIH effects. Here, we investigated whether LDT and LHA neurons projecting to the VTA are activated by physical exercise in NPP model mice.

## Results

**Figure 1:**
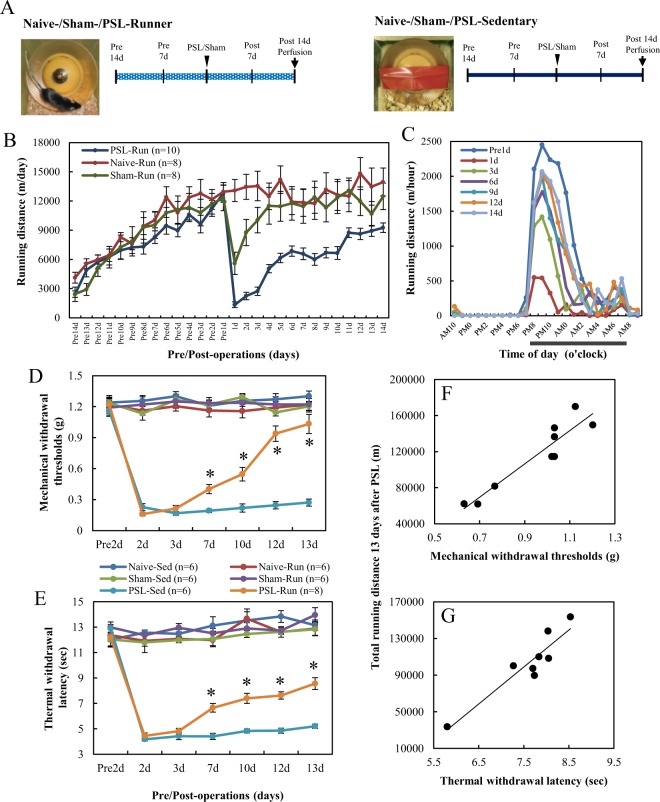
Changes in running distances and pain behaviors during the VWR period. (**A**) Protocol of VWR. Naive-, Sham- and PSL-Runner mice were allowed to run freely on the running wheel, whereas Sedentary mice were reared in cages with a locked running wheel. Naive-Runner, Sham-Runner and PSL-Runner mice were placed in individual cages equipped with a low-profile wireless mouse running wheel. (**B**) Changes in running distances by Runner mice. (**C**) Changes in diurnal variation in running distances by PSL-Runner mice (n = 7). (**D**) von Frey and (**E**) plantar tests were performed for all experimental groups. Mechanical withdrawal thresholds and thermal withdrawal latencies were significantly higher for PSL-Runner mice than for PSL-Sedentary mice (*p < 0.01). Significant positive correlations were observed between total running distances during the 13-day period after PSL, and (**F**) the withdrawal threshold (R = 0.934, p < 0.001, n = 9) and (**G**) withdrawal latency (R = 0.929, p < 0.001, n = 8).

As shown in Fig. 1A, Naive-, Sham-, and partial sciatic nerve ligation (PSL)-Runner mice were allowed to run freely on the running wheel, while Sedentary mice were reared in cages with a locked running wheel. Mice were divided into six groups: (1) Naive-Sedentary mice: mice without any artificial surgery, (2) Naive-Runner mice: mice with VWR without any artificial surgery, (3) Sham-Sedentary mice: mice without VWR after sham surgery, (4) Sham-Runner mice: mice with VWR after sham surgery, (5) PSL-Sedentary mice: mice without VWR after PSL, and (6) PSL-Runner mice: mice with VWR after PSL.

### Changes in running distances and pain behaviors

The manner of exercise by experimental animals is classified into forced exercise, such as treadmill running and swimming, and voluntary exercise such as free running on a running wheel. The effectiveness of forced exercise in producing EIH effects has been demonstrated in many studies^3^. However, forced exercise induces several physiological adaptations similar with chronic stress^21^, whereas VWR produces not only EIH effects in NPP, inflammatory pain and chronic muscle pain model animals, but also improves symptoms of chronic stress such as depression and anxiety-like behaviors^22–28^. In addition, VWR can be performed in the active phase during night without perturbing the rest phase during daytime. Thus, VWR is a stress-free exercise style, which may be more effective in producing EIH effects. Therefore, the present study used VWR and investigated EIH effects.

Figure 1B shows changes in running distances per day by Naive, Sham, and PSL**-**Runner mice. The mean running distance in the three groups was approximately 3000 m/day on day 1 after the start of the experiment (Pre14 d), and running distances markedly increased by day 14 (Pre1 d) (Naive-runner: 12948.03 ± 943.77 m/day; Sham-Runner: 12314.39 ± 1304.08 m/day; PSL-Runner: 12766.73 ± 886.59 m/day). The running distance by Sham-Runner mice decreased to 5591.72 ± 1147.08 m/day on day 1 post-surgery (1 d), but returned to nearly pre-surgery levels by day 4 post-surgery (11503.76 ± 1887.23 m/day: 93.4%). On the other hand, the running distance by PSL-Runner mice markedly decreased on day 1 post-surgery (1337.68 ± 286.54 m/day: 10.5%). However, these values gradually increased thereafter. The running distance by PSL-Runner mice recovered to approximately 72% (9250.59 ± 488.29 m/day) of the pre-surgery level by day 14 post-surgery (14 d). These changes were consistent with our preliminary results^19^. We then examined the diurnal variation in running distances (m/hour) by PSL-Runner mice (Fig. 1C). The peak running distance by Runner mice at day 1 pre-surgery (Pre1 d) was approximately 2449.52 ± 167.72 m/hour, but at day 1 post-surgery (1 d), it markedly decreased to 541.03 ± 238.95 m/hour. Then, the running distances recovered day by day after surgery, and at day 14 post-surgery (14 d), the peak running distance recovered to approximately 84.3% (2066.96 ± 66.89 m/hour) of the pre-surgical level.

We performed von Frey and plantar tests to evaluate the effects of VWR on mechanical allodynia and heat hyperalgesia (Fig. 1D,E). No marked differences were noted in the pre-operative data of withdrawal thresholds or withdrawal latencies among mice assigned to the six experimental groups. Furthermore, Runner mice in the Naive and Sham groups exhibited no significant alterations in withdrawal thresholds or withdrawal latencies throughout the experimental period, indicating that VWR had no effects on sensory sensitivity in mice without PSL. PSL-Sedentary mice had markedly lower withdrawal thresholds (0.227 ± 0.035 g) and shorter withdrawal latency (4.17 ± 0.137 sec) at 2 days after PSL than at pre-surgery (withdrawal threshold: 1.16 ± 0.052 g; withdrawal latency: 12.9 ± 0.307 sec), and these decreased thresholds and latencies were maintained throughout the experimental period. Withdrawal thresholds and latencies were significantly higher and longer on day 7 in PSL-Runner mice (0.402 ± 0.044 g, p < 0.01; 6.65 ± 0.342 sec, p < 0.01) than in PSL-Sedentary mice (7 days = 0.192 ± 0.017 g; 7 days = 4.4 ± 0.245 sec). Furthermore, significant increases in withdrawal thresholds and latencies were observed until day 13 after PSL in PSL-Runner mice compared with those in PSL-Sedentary mice (p < 0.01). Taken together with our previous findings^19^, the present results confirmed that the pain behaviors in NPP model mice were significantly improved by the VWR protocol employed in the present study. We next examined the relationships between the threshold of final pain on behavioral tests (13 d) and total running distances during the 13-day period after PSL in PSL-Runner mice. We found a significant positive correlation between total running distance, and withdrawal threshold (Fig. 1F; R = 0.934, p < 0.001, n = 9) and withdrawal latency (Fig. 1G; R = 0.929, p < 0.001, n = 8). These results also confirmed our previous findings^19^.

### VWR/PSL-induced changes in lVTA-dopamine neurons

As TH is a rate-limiting enzyme for dopamine synthesis, immunostaining with a TH antibody enabled identification of DA neurons in brain sections. In addition, immunoreactivity of FosBΔFosB was used as a marker of activated neurons because FosB protein gradually accumulates in response to chronic stimuli, including wheel running, and persists for long periods of time because of its high stability^16^. The VTA, which is composed of anatomically and functionally heterogeneous DA neuron subpopulations with different axonal projections, is divided into two parts: the lateral VTA (lVTA) and medial VTA (mVTA)^5^. A previous study suggested that mVTA-DA neurons projecting to the mPFC may be the primary subpopulation of DA neurons that are preferentially activated by aversive stimuli, including chronic pain, whereas lVTA-DA neurons projecting to the NAc lateral shell may primarily signal reward and salience^29^. Therefore, we focused on lVTA-DA neurons, and examined the effects of PSL and VWR on these neurons.

**Figure 2:**
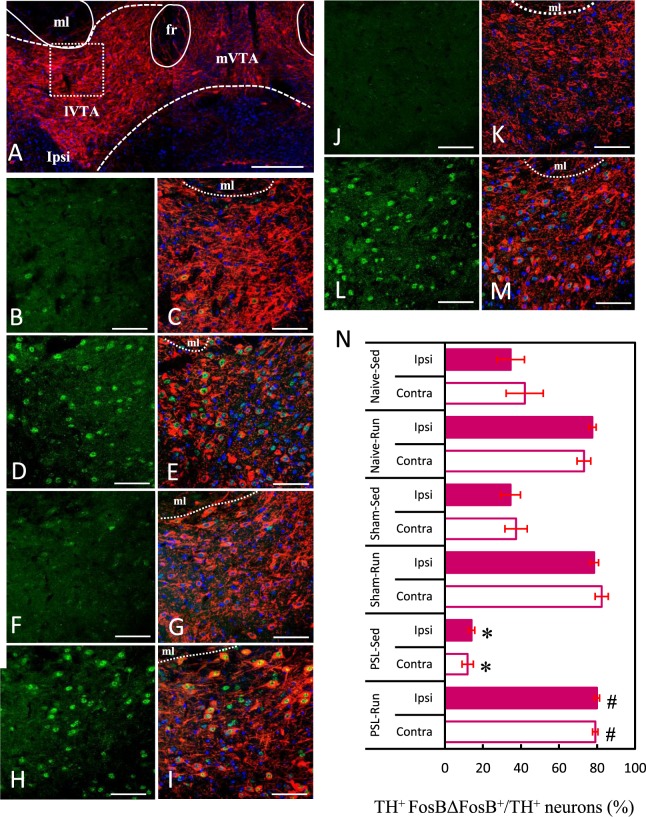
VWR/PSL-induced changes in lVTA-dopamine neurons. (**A**) Photomicrographs showing the anatomical region of lVTA with TH immunostaining (red). ml: medial lemniscus, fr: fasciculus retroflexus, lVTA: lateral VTA, mVTA: medial VTA. Photomicrographs show activated dopamine neurons in the lVTA of (**B**,**C**) Naive-Sedentary, (**D**,**E**) Naive-Runner, (**F**,**G**) Sham-Sedentary, (**H**,**I**) Sham-Runner, (**J**,**K**) PSL-Sedentary and (**L**,**M**) PSL-Runner mice. (**B**,**D**,**F**,**H**,**J** and **L**) indicate FosBΔFosB^+^ immunoreactivity, whereas (**C**,**E**,**G**,**I**,**K** and **M**) indicate dual immunostaining (FosBΔFosB^+^ TH^+^ DAPI). Bars: (**A**) = 200 μm, (**B**–**M**) = 70 μm. (**N**) A bar chart showing the proportion of activated dopamine neurons (TH^+^ FosBΔFosB^+^/TH^+^) in the lVTA in each group. As shown in (**A**) a square of 200 μm × 200 μm in size was placed on the lVTA in microscopy images, and TH^+^ FosBΔFosB^+^ cells within were counted. PSL significantly decreased the proportion of activated lVTA-DA neurons compared with that in other groups (n = 6, *p < 0.01 vs other groups), whereas VWR significantly increased the proportion of activated lVTA-DA neurons compared with that in other groups (n = 6, ^#^p < 0.01 vs all sedentary groups).

Figure 2A shows the position of lVTA on photomicrographs, which confirmed the report of Lammel *et al*.^5^. Figure 2B–M show typical immunostained images of activated lVTA-DA neurons in each experimental group, and the proportions of activated DA neurons in the lVTA on the ipsilateral and contralateral sides were compared among each group (Fig. 2N). The square in Fig. 2A indicates the area of lVTA in which the quantitative analysis was made. In agreement with the photomicrographs, the proportions of activated lVTA-DA neurons (TH^+^ FosBΔFosB^+^/TH^+^) were significantly higher in Naive-, Sham- and PSL-Runner mice than those in Naive- and Sham-Sedentary mice (p < 0.01). In contrast, the proportion of activated lVTA-DA neurons in PSL-Sedentary mice were significantly lower compared with those in the other groups (p < 0.01). There were no significant differences between the ipsilateral and contralateral sides in all groups.

### VWR/PSL-induced changes in LDT-cholinergic neurons

**Figure 3:**
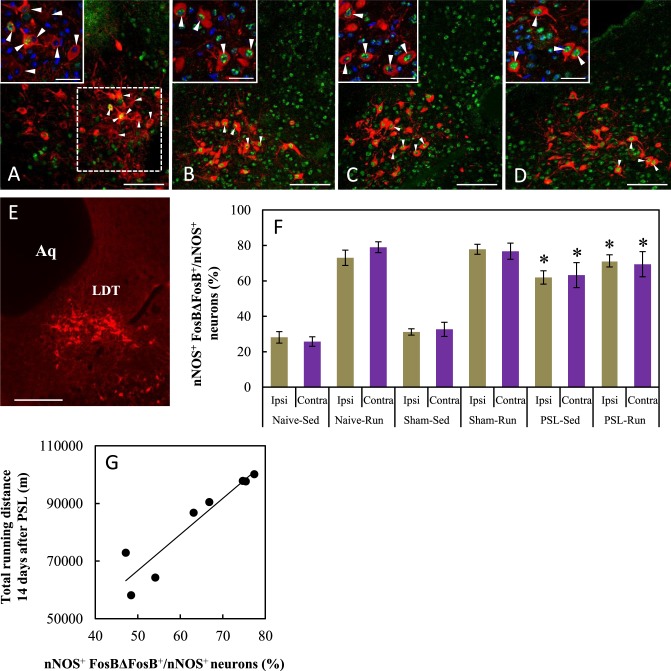
VWR/PSL-induced changes in LDT-cholinergic neurons. Photomicrographs show immunoreactivity of nNOS (Red) and FosBΔFosB (Green) on the contralateral side of the LDT in (**A**) Sham-Sedentary, (**B**) Sham-Runner, (**C**) PSL-Sedentary and (**D**) PSL-Runner mice. Neurons marked with arrowheads are enlarged in the insets in the upper-left corner (nNOS (Red), FosBΔFosB (Green) and DAPI (blue)). (**E**) Photomicrographs showing cholinergic neurons in the LDT with nNOS immunostaining (red). fr: Fasciculus retroflexus. Aq: aqueduct. LDT: laterodorsal tegmental nucleus. Bars: (**A**–**D**) = 100 μm, Inset = 30 μm, (**E**) = 200 μm. (**F**) A bar chart showing the proportion of activated cholinergic neurons (nNOS^+^ FosBΔFosB^+^/nNOS^+^) in the LDT in each group. As shown in (**A**) a square of 200 μm × 200 μm in size was placed on the LDT on microscopy images, and nNOS^+^ FosBΔFosB^+^ neurons within were counted. VWR and PSL significantly increased the proportion of activated LDT-cholinergic neurons compared with that in Naive- and Sham-Sedentary mice (n = 6, *p < 0.01). (**G**) A significant positive correlation was noted between total running distances during the 14-day period after PSL and the proportion of activated LDT-cholinergic neurons in PSL-Runner mice (R = 0.939, n = 8, p < 0.001).

The LDT contains three distinct neuronal types, cholinergic, GABAergic and glutamatergic neurons^30^. In the present study, immunostaining with a nNOS antibody was used to identify cholinergic neurons in brain sections because nNOS and choline acetyltransferase are useful target proteins for the immunohistochemical identification of cholinergic neurons in the mouse LDT^31^. Using dual immunostaining with nNOS and FosBΔFosB antibodies, we found many nNOS^+^ neurons without FosBΔFosB^+^ immunoreactive nuclei on both the ipsilateral and contralateral sides of the LDT in Sham-Sedentary mice (Fig. 3A). However, robust immunoreactivity of FosBΔFosB was bilaterally detected in nNOS^+^ neurons located in the LDT in Sham-Runner, PSL-Sedentary and PSL-Runner mice (Fig. 3B–D). The proportions of activated cholinergic (nNOS^+^ FosBΔFosB^+^/nNOS^+^) neurons on the ipsilateral and contralateral sides in the LDT were compared in Fig. 3F. Consistent with the immunostaining images, the proportions were significantly higher in all Runner mice and PSL-Sedentary mice than in Naive- and Sham-Sedentary mice (p < 0.01). No significant differences between the ipsilateral and contralateral sides were observed in all groups.

When the relationship between the total running distance during the 14-day period after surgery in PSL-Runner mice and the proportion of activated cholinergic neurons (sum of both the ipsilateral and contralateral sides) in PSL-Runner mice was examined, a significant positive correlation was noted (Fig. 3G, R = 0.939, n = 8, p < 0.01).

### lVTA-projecting LDT cholinergic neurons in Runner mice

**Figure 4:**
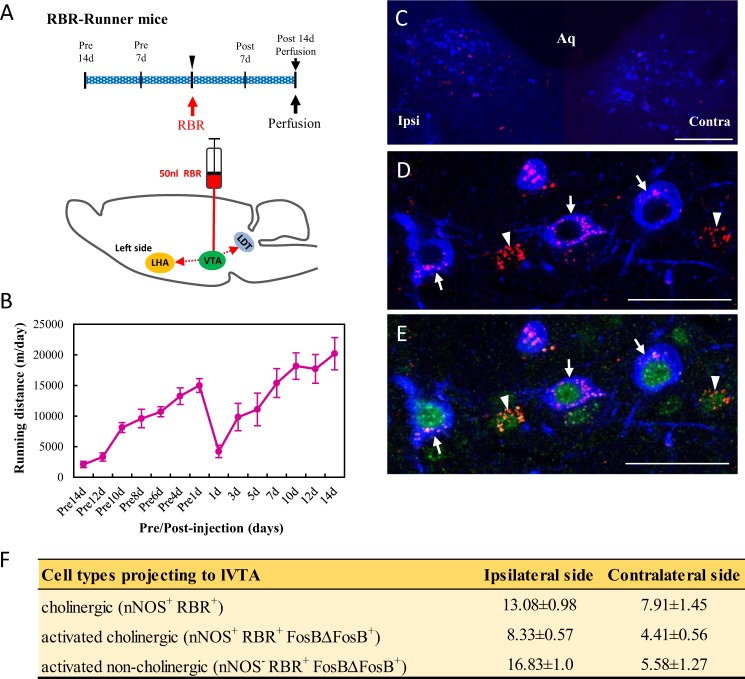
lVTA-projecting LDT cholinergic neurons in Runner mice. (**A**) After two weeks of VWR, 50 nl of RBR was injected into the left side of the lVTA in mice, and RBR-injected mice continued VWR for 2 more weeks. (**B**) Changes in running distances in RBR-injected mice (n = 6). (**C**) Photomicrograph showing positive signals for RBR (Red) and nNOS (Blue) in the ipsilateral and contralateral sides of the injection in the LDT. Aq: aqueduct. (**D**,**E**) Photomicrographs showing positive signals for nNOS (Blue), RBR (Red) and FosBΔFosB (Green) in the LDT. Arrows and arrowheads indicate lVTA-projecting activated LDT-cholinergic (nNOS^+^ RBR^+^ FosBΔFosB^+^) neurons and lVTA-projecting activated LDT-non-cholinergic (nNOS^−^ RBR^+^ FosBΔFosB^+^) neurons, respectively. Bars: (**C**) = 50 μm, (**D**,**E**) = 20 μm. (**F**) A table showing the quantitative results of cholinergic, lVTA-projecting activated cholinergic and lVTA-projecting activated non-cholinergic neurons on the ipsilateral and contralateral sides of the injection (n = 6). Quantitative data are presented as the mean ± standard error of the mean (SEM).

Using retrograde tracing and dual immunostaining, we investigated whether lVTA-projecting LDT-cholinergic neurons are activated by VWR. Mice were placed in individual cages with a running wheel at the start of the experiment, and after 2 weeks (Pre14 d), the running distance increased to 14999.47 ± 1124.61 m/day, as shown in Fig. 4B. A retrograde tracer, Retrobeads Red (RBR), was unilaterally injected into the left side of the lVTA, as described in Methods, and the RBR-injected mice then continued VWR for 2 weeks (Fig. 4A,B). Although the injection of RBR markedly decreased the running distance at day 1 post-injection (1 d: 4214.88 ± 1021.74), the running distances gradually increased thereafter until day 14 post-injection (14 d: 20193.53 ± 2633.39 m/day) (Fig. 4B). The brainstems in six mice in which RBR was precisely injected into the lVTA were used for dual immunostaining.

Although RBR-incorporated neurons were bilaterally detected in the LDT (Fig. 4C), more RBR^+^ nNOS^+^ neurons (arrows in Fig. 4D) were detected on the ipsilateral side (13.08 ± 0.98) than on the contralateral side (7.91 ± 1.45) of the injection (Fig. 4F). These results were consistent with previous findings, which demonstrated that following the unilateral injection of Fluoro-Gold or RBR into the VTA, the positive labeling of tracers was bilaterally detected in LDT-cholinergic neurons; however, the amount on the ipsilateral side was higher than that on the contralateral side^32,33^. On dual immunostaining with nNOS and FosBΔFosB antibodies, we found two distinct neuronal types, namely, lVTA-projecting activated cholinergic (nNOS^+^ RBR^+^ FosBΔFosB^+^) and lVTA-projecting activated non-cholinergic (nNOS^−^ RBR^+^ FosBΔFosB^+^) neurons (Fig. 4E). In the quantitative analysis, many lVTA-projecting activated non-cholinergic and lVTA-projecting activated cholinergic neurons were detected on the ipsilateral and contralateral sides, and the number of lVTA-projecting activated non-cholinergic neurons was approximately 2-fold that of the lVTA-projecting activated cholinergic neurons on the ipsilateral side (Fig. 4F).

### VWR/PSL-induced changes in LH-orexin neurons

**Figure 5:**
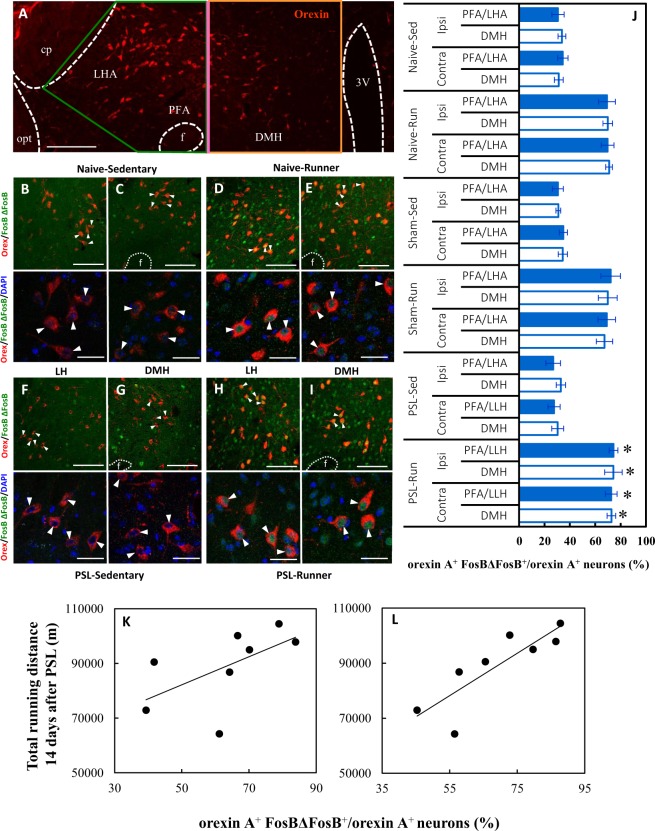
VWR/PSL-induced changes in LH-orexin neurons. (**A**) A photomicrograph showing anatomical regions of PFA/LHA and DMH, and orexin immunoreactivity (red). The vertical bar (pink) indicates the boundary between the DMH and PFA/LHA. cp: cerebral peduncle, opt: optical tract, f: fornix, PFA: perifornical area, LHA: lateral hypothalamic area, DMH: dorsomedial hypothalamus, 3V: 3rd ventricle. (**B**–**I**) The upper photomicrographs indicate activated orexin neurons (orexin A^+^ FosBΔFosB^+^). The activated orexin neurons marked with arrowheads in the upper panels are enlarged in the lower panels (arrowheads: orexin A^+^ (red) FosBΔFosB^+^ (green) DAPI^+^ (blue)). Photomicrographs show activated DMH and PFA/LAH-orexin neurons in (**B**,**C**) Naive-Sedentary, (**D**,**E**) Naive-Runner, (**F**,**G**) PSL-Sedentary, (**H**,**I**) PSL-Runner. Bars: (**A**) = 200 μm, (**B**–**I**) = 30 μm. (**J**) A bar chart showing the proportion of activated orexin neurons (orexin A^+^ FosBΔFosB^+^/orexin A^+^) in the PFA/LLH and DMH in each group. As shown in (**A**) the LH was divided into the PFA/LLH and DMH by a vertical bar, and orexin A^+^ FosBΔFosB^+^ cells within were counted. The proportions of activated orexin neurons in both the PFA/LLH and DMH were significantly increased compared with those in Naive-, Sham- and PSL-Sedentary mice (n = 6, *p < 0.01 vs all Sedentary groups). A significant positive correlation was detected between total running distances during the 14-day period after PSL and the proportion of activated orexin neurons (orexin A^+^ FosBΔFosB^+^/orexin A^+^) in PSL-Runner mice in (L) the PFA/LHA (R = 0.846, n = 8, p < 0.01), but not in (K) the DMH (R = 0.585, n = 8, NS).

Orexins are hypothalamic neuropeptides comprising orexin A and orexin B that are involved in multiple physiological processes such as the regulation of the sleep/wakefulness state, energy homeostasis, analgesia and reward seeking. Orexins bind to the orexin receptor type 1 (OR1) and orexin receptor type 2 (OR2), which are expressed on VTA neurons^34^. Orexin neurons are localized exclusively in the lateral hypothalamus (LH), including the lateral hypothalamic area (LHA), perifornical area (PFA) and dorsomedial hypothalamus (DMH), but their projections are widely distributed over many brain areas^34^. As shown in Fig. 5A, the LH was divided into two regions, the DMH and PFA/LHA, according to a criterion described in the Methods section. Orexin neurons arising in the DMH and PFA project to brainstem nuclei and are involved in the control of arousal and modulation of stress responses, whereas neurons in the LHA project to brain areas, including the VTA and NAc, and modulate reward and addiction processes^35,36^. However, as the PFA is anatomically small and disappears in the rostral part, we considered PFA/LHA-orexin neurons to mainly regulate reward processing in this study.

Using dual immunostaining with orexin A and FosBΔFosB antibodies, we found many orexin A^+^ neurons with strongly FosBΔFosB^+^ immunoreactive nuclei in both the DMH and PFA/LHA in Naive- and PSL-Runner mice (Fig. 5D,E,H,I), whereas many orexin A^+^ neurons lacking FosBΔFosB^+^ immunoreactivity were detected in both regions in Naive- and PSL-Sedentary mice (Fig. 5B,C,F,G). Furthermore, the intensity of orexin A immunoreactivity was very weak in Sedentary (Fig. 5B,C,F,G) compared with that in Runner mice (Fig. 5D,E,H,I). Based on the photomicrographs, the proportion of activated orexin neurons (orexin A^+^ FosBΔFosB^+^/orexin A^+^) was significantly higher in Naive-, Sham- and PSL-Runner mice than that in Sedentary mice (n = 6, p < 0.01) (Fig. 5J). No significant differences between the ipsilateral and contralateral sides were observed in all groups.

When the relationships between total running distances during the 14-day period after PSL and the proportions of activated orexin neurons in PSL-Runner mice (sum of both the ipsilateral and contralateral sides) were examined, a significant positive correlation was detected between total running distance and the proportion of activated orexin neurons in the PFA/LHA (R = 0.846, n = 8, p < 0.01) (Fig. 5L) but not in the DMH (R = 0.585, n = 8, NS) (Fig. 5K).

### lVTA-projecting orexin neurons in Runner mice

**Figure 6:**
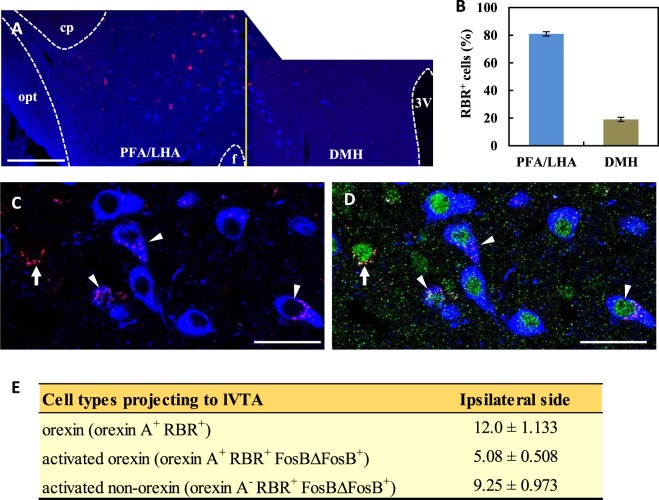
lVTA-projecting orexin neurons in Runner mice. (**A**) Photomicrographs showing RBR signals (red) and orexin immunoreactivity (blue) in the lateral hypothalamus on the ipsilateral side of the injection. cp: cerebral peduncle, opt: optical tract, f: fornix, PFA: prefrontal area, LHA: lateral hypothalamic area, DMH: dorsomedial hypothalamus, 3V: 3rd ventricle. The vertical line (yellow) indicates the boundary between the DMH and PFA/LHA. (**B**) Distribution of RBR^+^ cells in the lateral hypothalamus (n = 6). More RBR^+^ cells were detected in the PFA/LHA than in the DMH. (**C**,**D**) Photomicrographs showing positive signals for orexin A (Blue), RBR (Red) and FosBΔFosB (Green) in the LHA. Arrows and arrowheads indicate lVTA-projecting activated PFA/LHA-orexin (orexin A^+^ RBR^+^ FosBΔFosB^+^) neurons and lVTA-projecting activated PFA/LHA-non-orexin (orexin A^−^ RBR^+^ FosBΔFosB^+^) neurons, respectively. Bars: (**A**) = 200 μm, (**C**,**D**) = 30 μm. (**E**) A table showing the quantitative results for PFA/LHA-orexin, lVTA-projecting activated PFA/LHA-orexin and lVTA-projecting activated PFA/LHA-non-orexin neurons on the ipsilateral side of the injection site (n = 6). Quantitative data are presented as the mean ± standard error of the mean (SEM).

We next investigated whether orexin neurons projecting to the lVTA are activated by VWR. The VWR protocol and retrograde tracing were the same for the LDT, as shown in Fig. 4A and B. Almost all RBR^+^ cells in the LH were detected on the ipsilateral side of the injection, although RBR^+^ cells were occasionally observed on the contralateral side (Fig. 6A). In the present study, structures in which at least five RBR particles were arranged in either a ring- or disk-like shape were considered to be RBR^+^ cells (e.g., arrows and arrowheads in Figs 4D and 6C). Most RBR^+^ cells (approximately 80%) were distributed in the PFA/LHA and the rest were found in the DMH (Fig. 6B), indicating that PFA/LHA-orexin neurons abundantly project to the lVTA. On dual immunostaining with orexin A and FosBΔFosB antibodies, we found two distinct neuronal types, namely, lVTA-projecting activated orexin (orexin A^+^ RBR^+^ FosBΔFosB^+^) and lVTA-projecting activated non-orexin (orexin A^−^ RBR^+^ FosBΔFosB^+^) neurons (Fig. 6C,D). Quantitative analysis indicated that the VWR activated approximately 42% of the lVTA-projecting orexin neurons, and the number of lVTA-projecting activated non-orexin neurons was approximately 1.8-fold greater than that of lVTA-projecting activated orexin neurons (Fig. 6E).

## Discussion

The ability of the VWR to produce EIH effects has been confirmed by recent experimental studies^22–24,37–40^. Consistent with these studies, our results demonstrated that VWR for 2 weeks before and after surgery significantly improves pain behaviors. Thus, VWR is a reliable exercise style to produce EIH effects, although the VWR protocols and possible mechanisms of EIH differ among studies. We found a significant positive correlation between the total running distance and threshold of pain on behavioral tests, implying that the amount of VWR is a critical factor governing the analgesic level of EIH effects. On the contrary, Grace *et al*.^23^ reported that VWR prior to chronic constriction injury in rats completely prevented the development of allodynia, but the running distance and pain threshold were not correlated. Similarly, Pitcher *et al*.^24^ found that VWR by inflammatory pain model rats improved pain behaviors, but the amount of VWR did not correlate with analgesic levels. These discrepancies may be due to the differences in experimental designs and animal species used. We used mice as experimental animals, whereas they used rats. The physical activity level of mice is much higher than that of rats, and more active VWR may lead to more effective EIH. In accordance with our preliminary results^19^, the present study confirmed that the decreased running distances in VWR by PSL-Runner mice markedly recover day by day. In addition, the diurnal variation in running distances also recovered to almost pre-surgical levels. VWR has been well-reported as a natural reward that can activate VTA-DA neurons and the mesolimbic reward system^14–17^. Therefore, the gradual increase of running distance in VWR may cause higher activation of the mesolimbic reward system, which may help reduce NPP-induced aversion.

**Figure 7:**
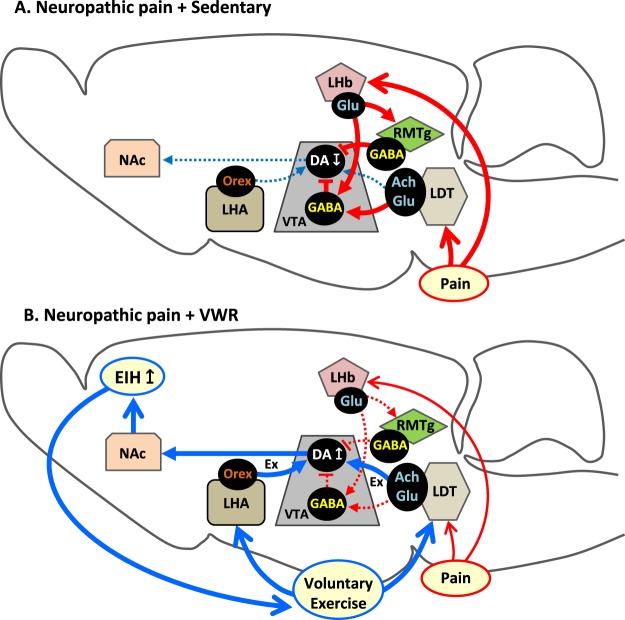
Potential mechanism of EIH effects following VWR. (**A**) In NPP + Sedentary mice, pain perception activates RMTg/VTA-GABA neurons via activation of LHb-glutamatergic neurons. LDT neurons also activate VTA-GABA neurons. Activated RMTg/VTA-GABA neurons inhibit VTA-DA neurons, which may promote aversion, depression and reduction of ADL. These changes may function to maintain and deteriorate NPP. (**B**) In NPP + VWR mice, activation of lVTA-DA neurons via activation of both LDT-cholinergic/glutamatergic neurons and LHA-orexin neurons enhances DA release into the NAc, which may activate the mesolimbic reward system, and thereby produce EIH effects. Creation of positive emotion and pleasure following the attenuation of pain perception reinforces the motivation to perform physical exercise, further enhancing the EIH effects.

Our results revealed that DA neurons in the lVTA are significantly inactivated in NPP model mice, which agrees with a recent finding that DA efflux in the NAc is reduced by painful stimulation^6^. It is well-documented that VTA-DA neurons are inactivated in painful conditions. Chronic pain or other aversive stimuli activate glutamate-containing LHb neurons, which then project to GABA neurons in the VTA and the more caudally located rostromedial tegmental nucleus (RMTg) to inhibit DA neurons in the VTA^5,41^. In addition to this LHb → RMTg/VTA (GABA) pathway, we cannot exclude the possibility that the direct LDT → VTA (GABA) pathway^42–45^ may also be involved in the inhibition of VTA-DA neurons and maintenance of NPP (Fig. 7A). Further studies are needed to elucidate the precise roles of this GABA neuron-targeted input from the LDT in the control of the reward system. On the other hand, we found that VWR significantly activates lVTA-DA neurons, and simultaneously enhances EIH effects in PSL-Runner mice. This is reasonable because many studies have demonstrated that VWR activates the mesolimbic reward system via VTA-DA neurons, and activation of the mesolimbic reward system induces analgesic effects^6,12,14–17,46^. Furthermore, Wakaizumi *et al*.^18^ demonstrated that specific inactivation of VTA-DA neurons attenuates the EIH effects induced by treadmill running in NPP model mice. In addition, based on our previous results, VWR significantly enhances TH immunoreactivity and pCREB expression in DA neurons in the lVTA^19^. Thus, activation of the mesolimbic reward system via activation of lVTA-DA neurons following VWR likely plays an important role in producing EIH effects.

We found that VWR significantly increases the proportion of activated LDT-cholinergic neurons. The lVTA-DA neurons receive direct cholinergic innervation from the LDT of the mesopontine tegmentum^33,47^, which regulates the activity of VTA-DA neurons. Efflux of DA in the NAc is stimulated by intra-VTA neostigmine, a cholinergic agonist, and this effect is blocked by excitotoxic lesions of the LDT^48^. Electrical stimulation of the LDT also increases NAc DA efflux through activation of nicotinic ACh and ionotropic glutamate receptors in the VTA^49^. Among the pontomesencephalic cholinergic neurons, VTA-projecting neurons are mostly concentrated in the LDT^33^ and these cholinergic neurons are noci-responsive^50^. It has been demonstrated that approximately 40% of them exhibit bursts of neuronal activity in response to noxious heat stimuli^50^. In the present study, approximately 60% of the LDT-cholinergic neurons on both sides were activated in PSL-Sedentary mice. On the other hand, 70∼80% of cholinergic neurons in the LDT were activated in Runner mice. Of note, similar proportions of LDT-cholinergic neurons (70%) were activated in PSL-Runner mice. As no additive effects were observed by concomitant PSL-surgery and VWR, the same neuronal group may respond to both PSL and VWR. Therefore, we speculate that these cholinergic neurons respond to arousal-related stimuli, such as pain, stress and exercise, and increase activity by controlling VTA-DA neurons for adaptation to new circumstances.

It has been reported that forced running by Parkinson’s disease model rats activates LDT neurons^51^. Using retrograde tracing and dual immunostaining, we found that VWR activated approximately 60% of the LDT-cholinergic neurons projecting to the lVTA on both sides in the present study. Therefore, based on the critical role of cholinergic innervation from the LDT for VTA-DA neurons^5,48^, our results suggest that VWR bilaterally activates LDT-cholinergic neurons, thereby activating lVTA-DA neurons. On the other hand, VWR also activated LDT-non-cholinergic neurons projecting to the lVTA, i.e., GABAergic and/or glutamatergic neurons. A previous study revealed that both LDT-cholinergic and LDT-glutamatergic inputs to the VTA contribute to rewarding effects, but they do so in different ways^52^. Therefore, both cholinergic and glutamatergic LDT neurons activated by VWR may produce EIH effects in different manners. However, the precise mechanisms need to be elucidated in more detail in future studies.

The present study demonstrated that VWR significantly increased the proportion of activated orexin neurons. Physical exercise has been reported to activate orexin neurons and increase orexin projection levels^53–55^. Upon investigation of the relationship between the proportion of activated orexin neurons and the total running distances after PSL, a significant positive correlation was detected for PFA/LHA-orexin neurons, but not for DMH. DMH-orexin neurons project preferentially to the locus ceruleus and are involved in arousal, whereas LHA-orexin neurons are mainly involved in reward and reinforcement of VTA-DA neurons^56^. Therefore, our results suggest that DMH-orexin neurons are activated in response to exercise irrespective of amount, and contribute to wakefulness, whereas LHA-orexin neurons enhance their activation levels in a VWR-dependent manner, which may activate lVTA-DA neurons and EIH.

According to retrograde tracing and dual immunostaining, the VWR activates lVTA-projecting orexin neurons. OR1 and OR2 are present in the VTA, and VTA-DA neurons receive excitatory inputs from LHA-orexin neurons^20,34^. Therefore, activation of lVTA-projecting LHA-orexin neurons following VWR is sufficient to activate lVTA-DA neurons. In addition, we found that VWR also activates lVTA-projecting non-orexin neurons. The LHA contains not only orexins and MCH, but also GABA neurons^57,58^, and optogenetic stimulation of LHA-GABA neurons projecting to the VTA produces reward-related behaviors^59,60^. In our preliminary experiment, we found that VWR does not induce expression of FosBΔFosB in MCH neurons (data not shown), suggesting that VWR-activated non-orexin neurons correspond to LHA-GABA neurons. Therefore, VWR may also activate LHA-GABA neurons in addition to LHA-orexin neurons, both of which project to the lVTA. Yazdi-Ravandi *et al*.^61^ demonstrated that the injection of orexin A into the rat VTA produced analgesic effects, but this effect was blocked by the injection of D1 and D2 receptor antagonists into the NAc. In inflammatory pain model rats, pharmacological activation of orexin neurons induces analgesia, and inhibition of orexin receptors in the VTA can abolish these analgesic effects^62^. Furthermore, selective ablation of orexin neurons produces pain-related behaviors, whereas pharmacological activation of orexin neurons induces analgesia^63^. Thus, the analgesic effects of orexin may be mediated via activation of the mesolimbic reward system through activation of VTA-DA neurons. Therefore, activation of lVTA-DA neurons via LHA-orexin neurons, and possibly LHA-GABA neurons, following VWR may produce EIH effects.

Based on the present study, we propose two potential neuronal mechanisms of lVTA-DA neuron activation following VWR. One is by cholinergic/glutamatergic neurons in the LDT and the other is by orexin neurons in the LHA. The former neurons can be activated by both VWR and PSL, whereas the latter neurons are activated by VWR but not by PSL. Furthermore, many of these neurons project to the VTA. Therefore, we conclude that both LDT (cholinergic/glutamatergic) → lVTA (DA) and LHA (orexin) → lVTA (DA) pathways are involved in the activation of the mesolimbic reward system, which may produce EIH effects. The creation of positive emotion and pleasure following attenuation of pain perception reinforces the motivation to perform physical exercise, which may further potentiate EIH effects (Fig. 7B). This virtuous cycle may help to maintain, improve and establish an active lifestyle, which further relieves chronic pain and improves QOL. Therefore, further studies focused on brain neuronal systems, including the mesolimbic reward system, will enhance our understanding of the neuronal mechanisms producing EIH effects, and lead to the development of novel therapeutic strategies to improve and prevent chronic pain.

## Methods

### Preparation of NPP model mice

Adult C57BL/6J mice were used in this study. NPP model mice were prepared by PSL^64^. Under deep anesthesia, approximately one-third to one-half of the sciatic nerve at the right mid-thigh level was tightly ligated with 8–0 silk sutures. Sham operations were performed using the same procedures described above excluding PSL. All experiments in this study were approved by the Animal Care Committee of Wakayama Medical University (Ref. No. 642). All experiments conformed to the National Institutes of Health Guide for the Care and Use of Laboratory Animals (NIH Publication No. 99-158, revised 2002).

### Experimental groups and the VWR protocol

Mice were divided into six groups: (1) Naive-Sedentary mice (n = 6), (2) Naive-Runner mice (n = 6~8), (3) Sham-Sedentary mice (n = 6), (4) Sham-Runner mice (n = 6~8), (5) PSL-Sedentary mice (n = 6~10) and (6) PSL-Runner mice (n = 6~10). All Runner and Sedentary mice were placed in individual cages equipped with a running wheel or locked running wheel under a 12/12-hour light-dark cycle, and Naive-, Sham- and PSL-Runner mice were allowed to run freely on the running wheel. The VWR protocol is shown in Fig. 1A. All mice, except for Naive mice, performed VWR for 2 weeks prior to the sham operation or PSL. PSL and Sham mice were returned to individual cages immediately after surgery, and running distances were measured during the 14-day period after the surgery. The rotations ran on the running wheel/hour were monitored using a magnetic reed switch attached to a computerized exercise-monitoring system (SOF-860 wheel manager software, MED Associates, Inc.), and daily running distances (m/day) and diurnal variation in running distance (m/hour) were calculated using the number of rotations on the running wheel.

### Evaluation of pain behaviors

In order to abolish diurnal variation in pain behaviors, von Frey and plantar tests were performed at 8:15 a.m. Prior to both tests, mice were placed in an acrylic glass enclosure with a wire mesh bottom (8.3 × 8.3 × 8.0 cm) and allowed to acclimate for 30 min. Mechanical allodynia was evaluated using withdrawal threshold upon von Frey monofilament stimulation to the plantar surface of the hindpaw (0.023, 0.0275, 0.068, 0.166, 0.407, 0.692, 1.202 and 1.479 g). The up-down method of von Frey monofilament testing was used in the present study, and the minimum pressure (g) that evoked a quick withdrawal response by stimulation with a thin von Frey monofilament was taken as the withdrawal threshold^65^.

Heat hyperalgesia was evaluated by the plantar test, which measures withdrawal latency (sec) to a radiant thermal stimulus delivered from beneath the glass floor to the plantar surface of the hindpaw (Plantar Test, Model7371, Ugo Basile, Comerio, Italy). The heat stimulus by the projector lamp bulb was focused on the plantar surface of the ipsilateral hindpaw, and the minimum time (sec) needed to evoke quick withdrawal of the paw by heat stimulation was taken as the withdrawal latency^66^. The von Frey and plantar tests were performed three times for each mouse, and mean values were considered as the withdrawal threshold or withdrawal latency, respectively. In all behavioral evaluations, the investigator was blinded to the experimental groups in order to avoid any bias.

### Immunofluorescence analysis

The primary antibodies were as follows: FosBΔFosB (rabbit polyclonal antibody, dilution 1:400, Santa Cruz Biotechnology, sc-48), tyrosine hydroxylase (sheep polyclonal antibody, dilution 1:200, Merck Millipore Corporation, Cat #AB1542), nNOS (neuronal) (goat polyclonal antibody, dilution 1:500, Abcam, ab1376) and orexin A (goat polyclonal antibody, dilution 1:400, Santa Cruz Biotechnology, sc-8070).

Mice were perfused transcardially with 4% paraformaldehyde in 0.1 M phosphate-buffered saline (PBS), and the brain was post-fixed. After cryoprotection, the brain was frozen using dry ice-cooled hexane. Serial 25-μm thick brain sections including the VTA (Bregma −3.52 mm~−3.08 mm), LDT (Bregma −5.02 mm~−4.84 mm) or LHA (Bregma −2.06 mm~−1.34 mm) in the coronal plane were mounted serially on slides. After blocking for non-specific staining, sections for dual immunofluorescence staining were incubated simultaneously with two kinds of primary antibodies diluted in 0.1 M PBS containing 5% normal donkey serum and 0.3% Triton X-100 at 4 °C for 48 hours. Then, sections were incubated with the secondary antibodies diluted in 0.1 M PBS containing 5% normal donkey serum and 0.1% Triton X-100 during overnight at 4 °C. The secondary antibodies used for the detection of TH, nNOS and orexin A immunoreactivity were Alexa Fluor 594-labeled donkey anti-sheep (1:500, Jackson ImmunoResearch Laboratories, Inc., Coad Number: 713-585-147) or Alexa Fluor 594-labeled donkey anti-goat IgG (1:500, Abcam, ab150136). The secondary antibody used for the detection of FosBΔFosB immunoreactivity was Alexa Fluor 488-labeled donkey anti-rabbit IgG (1:500, Abcam, ab150061). Sections were washed in 0.1 M PBS and mounted in Vectashield mounting medium with DAPI (H-1200, Vector Labs., Burlingame, CA). Fluorescence signals were detected using a confocal microscope (LSM700, Carl Zeiss, Oberkochen, Germany) equipped with an argon-helium laser. Negative control sections, which were processed without primary antibodies, had no significant positive immunoreactivity.

### Quantitative analysis of immunopositive neurons

Immunofluorescence images of lVTA or LDT on the ipsilateral and contralateral sides were acquired at a magnification of 20×. A square of 200 μm × 200 μm in size, as shown in Fig. 2A for lVTA and Fig. 3A for LDT, was placed on the microscope images using ImageJ software (version 1.48, National Institutes of Health, USA), and the number of immunopositive cells within was counted. In a previous study, the LH was further divided into three regions: DMH, PFA and LHA^30^. In this study, the LH was divided into two regions, DMH and PFA/LHA, by a pink vertical line along the medial edge of the fornix, as shown Fig. 5A. Immunofluorescence images of the DMH and PFA/LHA on the ipsilateral and contralateral sides were acquired at a magnification of 10×. The number of immunopositive cells within the yellow square (DMH) or green pentagon (PFA/LHA) on microscopy images was counted. The mean value of three randomly selected sections from six sections per brain was regarded as the value of immunopositive neurons per mouse. In order to avoid any bias in the results of the quantitative analysis, investigators were blinded to all experimental groups throughout quantitative analyses.

### Injection of retrograde tracer into the lVTA and dual immunofluorescence staining

Mice performed VWR for 2 weeks prior to injection of the retrograde tracer, and were then placed in a stereotaxic apparatus. Unilateral injection of Retrobeads Red (RBR) (50 nl, Lumafluor Inc., Naples, FL) was performed by inserting a microsyringe into the left side of the lVTA (Bregma: anteroposterior, −3.1 mm; mediolateral, 0.5 mm; and dorsoventral, −4.5 mm). RBR-injected mice were returned to their individual cages, and the running distance was recorded for 2 weeks before and after injection. The brains of RBR-injected Runner mice were processed in the same manner as described for the Immunofluorescence analysis. To evaluate RBR-injection sites, 100-μm thick brain sections including the lVTA were mounted serially, and after briefly rinsing with PBS, slides were mounted in Vectashield mounting medium with DAPI.

Except for secondary antibodies to detect nNOS and orexin A immunoreactivity, the same immunostaining procedure and quantitative analysis were adapted for RBR-injected brain sections. The secondary antibody used to detect specific signals for nNOS and orexin A was DyLight 405-labeled donkey anti-goat IgG (1:500, Jackson Immuno Research Laboratories, Inc.). The total number of cells in three randomly selected sections from six sections per RBR-injected Runner mouse was regarded as the value of immunopositive cells. In order to avoid any bias in the results of the quantitative analysis, investigators were blinded to all experimental groups throughout quantitative analyses.

### Statistical analysis

Quantitative data are presented as the mean ± standard error of the mean (SEM). A repeated measures ANOVA followed by Bonferroni’s post hoc test was used to compare withdrawal threshold and withdrawal latency among the experimental groups. Multiple comparisons of the number of immunopositive cells among experimental groups were performed using a one-way ANOVA followed by Bonferroni’s post hoc test. Differences were considered to be significant at p < 0.05.

### Data availability

All data generated or analysed during this study are included in this published article.
